# COGNITIVE DECLINE AMONG OLDER ADULTS IN RURAL CHINA: THE PROTECTIVE ROLE OF FORMAL SCHOOLING

**DOI:** 10.1093/geroni/igz038.745

**Published:** 2019-11-08

**Authors:** Yaolin Pei, Bei Wu, Zhen Cong, Mengyao Hu

**Affiliations:** 1 New York University, New York, New York, United States; 2 Rory Meyers College of Nursing, New York University, New York, New York, United States; 3 School of Social Work, University of Texas at arlington, Arlington, Texas, United States

## Abstract

Evidence shows that education is strongly associated with cognitive functioning; however, few studies have examined the effect of education on cognitive decline among older adults with very limited education. Our study analyzed six waves of panel data (2001, 2003 2006, 2009, 2012 and 2015) from the Longitudinal Study of Older Adults in Anhui Province, China. We estimated two-level multilevel models of cognitive functioning for older adults age 60+, sampled using probability sampling strategy. We found that having formal schooling was positively associated with better cognitive functioning. Older adults with formal schooling had slower decline in cognition and the gap in cognition between the literate and illiterate widened with age. These findings highlight the role of early life experience in affecting cognitive function in later life and suggest that disadvantages in cognitive functioning accumulate throughout the life course for persons with no formal education.

